# ESTSS and ISTSS: “heterozygous twins”

**DOI:** 10.3402/ejpt.v4i0.21307

**Published:** 2013-06-06

**Authors:** Berthold P. R. Gersons

**Affiliations:** Academic Medical Center, University of Amsterdam, Arq Psychotrauma Expert Group, Diemen, The Netherlands

**Keywords:** PTSD, ESTSS, ISTSS

## Abstract

The development of traumatic stress studies during the past decades has much profited from professionals from the United States and from Europe. However, these professional societies, the International Society for Traumatic Stress Studies and the European Society for Traumatic Stress Studies (ESTSS) still struggle to find an equal common pathway. This is a personal retrospective view of Berthold Gersons, past president of ESTSS on behalf of the 20th anniversary of ESTSS.

The celebration of 20 years of European Society for Traumatic Stress Studies (ESTSS) brings us back to 1993 when ESTSS was formally launched as the European Society for Traumatic Stress Studies in Bergen, Norway during the Third European Conference on Traumatic Stress. However, the First European Conference was already held in 1988 in Lincoln, UK. The roots of ESTSS started 25 years ago! (I)STSS was “born” in 1985 with Charles Figley as its first president (Bloom, 2000). On both sides of the Atlantic, the new concept of traumatic stress stimulated scientists, psychotherapists, and other practitioners to meet with each other at conferences and within newly founded societies. Because I have been involved in both societies for many years, I would like to pay attention to the important and sometimes complicated interactions between ESTSS and International Society for Traumatic Stress Studies (ISTSS) over the years.

## (I)STSS: the start

My first encounter with (I)STSS was in 1989 at the Fifth Annual Meeting of the Society for Traumatic Stress Studies (STSS) in San Francisco. I became familiar with STSS through three Dutch colleagues: *Wybrand Op den Velde*, psychiatrist in Amsterdam who held monthly lectures at his hospital in Amsterdam and who has been a pioneer on World War II studies in The Netherlands; *Wim Wolters*, child psychologist and my colleague professor at Utrecht University; and *Bessel van der Kolk* who was born in The Netherlands but became a psychiatrist in the United States, lives in Boston and is a pioneer on traumatic stress studies. I myself was trained as a psychiatrist and psycho-analyst in Amsterdam, but until 1990 was mainly active in the field of community psychiatry and prevention. Through my encounter with police officers in Amsterdam who developed PTSD after shooting incidents, I became interested in traumatic stress, in treatment of PTSD and in prevention (Gersons, 1989; Gersons, Carlier, Lamberts, & Van der Kolk, 2000). I will never forget the meeting in 1989 in San Francisco. The meeting became memorable because it took place 10 days after the devastating Pietro Loma earthquake, where 63 people were killed and nearly 4,000 were injured and many became homeless. The streets of San Francisco were filled with homeless people and highways and the Oakland Bay Bridge^1^ had collapsed. It became for me a double encounter with the science of traumatic stress and with the reality of a huge disaster. At the conference, many colleagues were active in supporting victims of the earthquake and also testifying their own personal experiences and fears. This helped me later when the El Al air crash disaster in 1992 struck the area next to our academic hospital in Amsterdam (Carlier & Gersons, 1997; Gersons & Carlier, 1993; Vermetten & Olff, 2013). The meeting in San Francisco was also the only one from ISTSS which paid attention to victim commemoration in the center of town at Union Square. This was a better initiative of Yael Danieli, the then ISTSS president.

In 1989, I was also asked by Charles Figley accompanied by Shad Mashed to organize the First World Conference on Traumatic Stress, to be held in Amsterdam in 1992 for STSS. Unaware of the competition between STSS and the pre-ESTSS group headed by Walter de Loos, I helped with people like Charles Figley and Yael Danieli who wanted to make their American organization an international one. Together with Jos Weerts and Carl Steinmetz, we organized a five-day conference with 700 participants from all over the world and with highlights like a pioneer panel with Leon Eitinger, Jan Bastiaans, Henry Krystal, and Lawrence Kolb, with the Dutch Queen at the opening ceremony and a reception with the famous painting, ‘Night Watch’ of Rembrandt in the background. I proposed to the STSS board to put an “I” in their name, to make it clear they wanted to be a real international society. However, because of the still hybrid character of ISTSS, the wish to be a serious international organization has been hampered always because in reality 80% of its members are from the United States. Also, after this first world conference in June 1992, their annual meeting was held on American soil in November of that year in Los Angeles. I was a member of the ISTSS board from 1996 to 1999 and I have been member of many program committees of ISTSS meetings and a two-times member of the editorial board of JTS. But ISTSS never became really an international organization. Board meetings were always dominated by topics important for the United States. Up until 1993, ISTSS followed a strategy to become recognized as an international umbrella organization by affiliating only Traumatic Stress Societies overlooking continents, such as the Australasian STSS and the ESTSS. After the Gulf War in 1993, ISTSS dropped this strategy by affiliating with the Kuwaiti SSTS. When the new German Speaking STSS (DEGPT) in 1998 was founded on their request, they were affiliated directly without asking for approval of the ESTSS.

## ESTSS

I was not involved with the 1988 Lincoln conference but in all other biannual ESTSS conferences^2^ I have been involved in different roles. I was asked to join the ESTSS Board in 1997, but I did not want to be a member of ESTSS and ISTSS at the same time. I joined the ESTSS Board from 1999 to 2008. As may be evident, I accepted the one-sided American interest in the ISTSS board because ISTSS has to serve its American members. ISTSS conferences are always held in similar big hotels in different US cities and a few in Canadian cities. It is a cost-effective formula because the big hotel is also the conference venue and it gives a free much-appreciated free suite for the president of the ISTSS. During the meeting, there is no connection with the city or the universities in that city.

ESTSS meetings differ so much in these aspects. Mostly, there is one conference venue and many hotels in the neighborhood. The meetings often start with live music from the country, a welcome from the minister or a local representative of the city council, the patronage of someone from the royalty or the president. Receptions are often in beautiful old town halls with much history, far away from anonymous hotels, with speeches from the city's government.

Of course, Europe is called the “old” continent where history is everywhere and where local culture, food, language, and pride but also much suffering are felt present. Roderick Orner also started a series of very successful local ESTSS meetings on early interventions. At the same time, the ESTSS Board could convene in those places where the ESTSS workshops were held.

However, this was more the “romantic” side of ESTSS. When I joined the board in 1999, it was Bas Schreuder who went to the notary to get official bylaws for ESTSS and to also start a small professional secretariat at his mental health organization Centrum 45 in the Netherlands. When Dean Ajdukovic took over the presidency from Bas, I became treasurer of a society that often did not receive any money from successful conferences and with a decreasing membership. There were no elections and the board was only renewed by cooptation. Under my presidency, we devised a strategic plan to change ESTSS into a large umbrella organization with local STSS in the different European countries. The first elections were held and candidates needed to present themselves with a bio and an idea on which contribution they wanted to make to ESTSS. Learning from ISTSS that a one-year presidency term is too short to consolidate developments, we chose a 4-year period, 1 year as president-elect, 2 years as president and 1 year as past-president. The presidents after me like Jon Bisson and Miranda Olff have implemented this strategy very successfully (see Bisson, 2013; Olff 2013). ESTSS is now as big as or even bigger than ISTSS.

## Conclusion

ESTSS and ISTSS were born in the same decade but with a different background and geopolitical orientation. They are also culturally different, i.e., ISTSS wants to be as scientific as possible while ESTSS acknowledge the value of narratives, oral histories, and not yet evidence-based practices. ESTSS biannual meetings are more visited by people from all over the world as the ISTSS meetings. This rivalry between ESTSS and ISTSS will continue and is to some extent productive. However, I am longing for a better balance in which ISTSS acknowledges their American membership and perhaps a new international umbrella can come into existence.

## Footnotes

1 This bridge was on the cover of the conference program intact.

2 Cities where ESTSS conferences have been held: 1988 Lincoln (UK), 1990 Noordwijkerhout (NL), 1993 Bergen (Norway), 1997 Maastricht (NL), 1999 Istanbul (Turkey), 2001 Edinburgh (UK), 2003 Berlin (Germany), 2005 Stockholm (Sweden), 2007 Opatija (Croatia), 2009 Oslo (Norway), 2011 Vienna (Austria), and 2013 Bologna (Italy).
